# Spatiotemporal Evolution and Influencing Factors of Carbon Emission Efficiency in the Yellow River Basin of China: Comparative Analysis of Resource and Non-Resource-Based Cities

**DOI:** 10.3390/ijerph191811625

**Published:** 2022-09-15

**Authors:** Yingqi Xu, Yu Cheng, Ruijing Zheng, Yaping Wang

**Affiliations:** College of Geography and Environment, Shandong Normal University, Jinan 250358, China

**Keywords:** carbon emission efficiency, resource-based cities, non-resource-based cities, super-efficiency SBM model, Yellow River Basin

## Abstract

Comparing the carbon emission efficiency (CEE) of resource and non-resource-based cities in the Yellow River Basin (YRB) can guide their synergistic development and low-carbon transition. This study used the super-efficiency slacks-based measure (super-SBM) model to measure the CEE of cities in the YRB. Kernel density estimation and Theil index decomposition methods were used to explore the spatiotemporal evolutionary patterns, and a panel regression model was established to analyze the influencing factors of CEE. The research results showed that the CEE of the two types of cities have an overall upward trend in time, with a widening regional gap. Resource-based cities mainly displayed the characteristics of decentralized regional agglomeration, while non-resource-based cities mainly showed the characteristics of convergent regional agglomeration. Panel regression results showed that the levels of economic development, indus-trial structure, and population density are significantly positively correlated with CEE in the YRB, while foreign direct investment and resource endowment are significantly negatively correlated with CEE. Except for economic development and industrial structure, there is some variability in the contribution of the remaining influencing factors to the CEE of the resource and non-resource-based cities. The research results suggest developing classification measures for low-carbon transition in the YRB.

## 1. Introduction

In the context of addressing climate change and promoting global climate governance, low-carbon transformation has become an important trend in the restructuring of the world economy. In 2019, the ecological protection and high-quality development of the Yellow River Basin (YRB) elevated to a major national strategy for China. Urban economic types can be divided into resource-dependent and non-resource-dependent, and the corresponding city types are resource-based cities and non-resource-based cities, respectively. Resource-based cities take the exploitation and processing of natural resources such as minerals and forests in the region as the leading industry, and the production and development of the cities are closely related to the development of resources. Both types of cities are widely distributed in China, and at present, more than half of the cities in this basin are resource-based cities. With continued industrialization, energy over-exploitation in resource-based cities in the YRB has led to a rapid rise in their carbon emissions, increasing their contribution to climate change problems significantly. At the same time, differences in economic development patterns and resource endowments between resource and non-resource-based cities in the basin are widening. The ability to develop green and low-carbon cities is related to the successful construction of a regional ecological civilization. Therefore, understanding the influencing factors of the carbon emission efficiency (CEE) of cities in the YRB and the realization of synergistic and efficient development are important ways to achieve regional low-carbon development. These are also top priorities to promote high-quality economic development in the YRB.

At present, scholars have focused on the environmental protection and high-quality development of the YRB in terms of industrial transformation [1,2], water resources utilization [3,4] land use [5,6], and the atmosphere [7,8], the latter focusing on haze pollution and carbon emissions. Carbon emissions research mainly includes total carbon emissions [9], intensity [10], structure [11], efficiency [12] and other research aspects.

Research on CEE in China and abroad focuses on the following aspects: First, Evaluation measures are divided into two categories: single-factor and, increasingly, multi-factor. Single-factor indicators are mostly measured as the ratio of carbon emissions to economic and energy-related indicators. Multi-factor indicators are measured with parametric and non-parametric methods [13]. The parametric method mainly uses the stochastic frontier approach (SFA), which is primarily used for single output and multi-input efficiency measurements [14,15]. For example, Wang et al. (2019) used the SFA model to measure the carbon emission performance of the industrial sector in the Beijing-Tianjin-Hebei region of China [16]. The non-parametric method, also known as the mathematical method, is commonly used for data envelopment analysis (DEA). The DEA, however, has poor accuracy of data measurement due to the slack variable problem so, at this stage, it has been replaced by the SBM-DEA model [17], super-efficiency SBM model [18], and EBM-DEA model with undesirable outputs [19] to measure the CEE. Second, For the characterization of the spatial and temporal evolution of CEE, studies mainly use the Theil index [20], Dagum Gini coefficient [21], coefficient of variation, kernel density estimation [22], and GIS spatial analysis. Some scholars applied spatial autocorrelation models and spatial Markov transfer matrix to reveal the spatial effects of carbon emission and found that the CEE of Chinese cities has significant spatial agglomeration and spatial spillover effects [23,24]. Finally, In terms of carbon emission influencing factors, geographical detectors, exponential decomposition method (e.g., Logarithmic Mean Divisia Index method) [25], and panel regression [26] are mainly used to identify and analyze the impact of socio-economic development factors on carbon emissions from different perspectives. Improvements in the economic development level [27] and optimization of the industrial structure [28] are conducive to improving CEE. Technological innovation can effectively improve CEE by improving the efficiency of energy resource utilization [29]. The effect of urbanization-induced population agglomeration on CEE follows a U-shaped curve [14], and foreign direct investment affects CEE through scale, structural and technological effects [30]. In addition, low-carbon policies [31], environmental regulation [32], land use [33], and the digital economy [34] have also been identified as important factors affecting CEE.

Although many theoretical and empirical studies have been made in the related studies of CEE, there are still a series of problems that need to be solved. First, existing studies mainly focused on the macro level, such as the national or provincial level [20,35]. However, less research has been done on CEE at the special scale spatial unit of the river basin. The importance of the study of the YRB is further emphasized by its dominance by traditional resource-intensive industries [36]. Second, the Theil index has been widely used to study CEE differences [35], however, the differences between and within groups are less frequently compared. Third, an overall regression analysis is often conducted based on all study samples to explore the main influencing factors, neglecting the heterogeneity of the role of influencing factors on different types of cities, and the reasons for the differences in carbon emission efficiency are not investigated. This study attempts to compare the CEE of YRB to answer the following three questions: (1) What are the similarities and differences in the spatial and temporal distribution patterns of the CEE of resource-based and non-resource-based cities in the YRB? (2) Where do the regional differences in CEE in the YRB come from? (3) Are there significant differences in the effects of various factors on the CEE of resource-based and non-resource-based cities? On this basis, this study used the super-efficiency SBM model to calculate the CEE of the YRB and the Theil index decomposition method to reveal the sources of spatial differences in the YRB. It finally compared and analyzed the influencing factors of CEE in the YRB and its resource and non-resource-based cities using a panel regression model to provide guidance on how to promote the low-carbon transition in the region.

This study contributes to the literature in the following ways: (1) Under the total factor framework and based on the input-output perspective, the super-efficiency SBM model considering undesirable output was introduced to measure the CEE, which addresses the problem that it is impossible to compare multiple effective DMUs when the calculation results of the traditional DEA model show that multiple effective DMUs are 1. (2) Considering the great differences between resource-based and non-resource-based cities in terms of resource endowments and economic development patterns, the YRB is divided into these two groups of cities for comparison, and the Theil index decomposition method is used to compare the differences in CEE between and within groups. (3) Based on the overall regression analysis of the study sample to explore the main influencing factors of CEE, the heterogeneity of the role of influencing factors on resource-based and non-resource-based cities is investigated. The remainder of this study is organized as follows: Section 2 describes the research area, research methods, and data sources; Section 3 presents the analysis results; Section 4 includes conclusions and policy suggestions.

## 2. Materials and Methods

### 2.1. Study Area and Data Source

**Figure 1:**
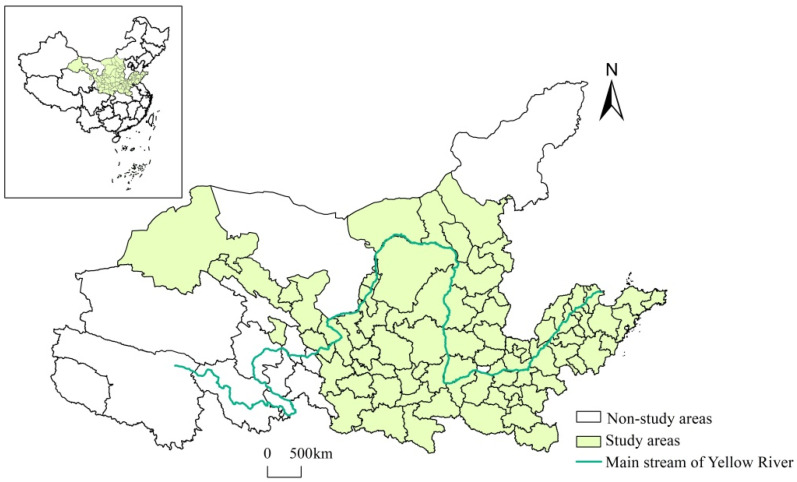
Scope of the Yellow River Basin.

Considering that Sichuan Province has been incorporated into the Yangtze River Economic Belt and that the four cities (leagues) of Hulun Buir, Chifeng, Tongliao, and Hinggan League in eastern Inner Mongolia belong to the northeast, the YRB in this study includes eight provincial administrative regions, including Qinghai, Gansu, Ningxia, Inner Mongolia (excluding the eastern three cities and a league), Shaanxi, Shanxi, Henan, and Shandong, with a total of 79 cities at the prefecture level and above in the YRB (Figure 1). According to the National Sustainable Development Plan for Resource-based Cities (2013–2020), 41 resource-based cities and 38 non-resource-based cities were selected from the 79 cities. The socio-economic data were mainly obtained from the China City Statistical Yearbook, China City Construction Statistical Yearbook, China Regional Economic Statistical Yearbook, and the statistical yearbooks of prefecture-level cities. Missing data of individual cities and individual years were interpolated. Carbon emission data were obtained from the Carbon Emission Accounts and Datasets (www.ceads.net) and city green patents granted were obtained from the patent search database of the State Intellectual Property Office of China (SIPO).

### 2.2. CEE Measurement

Data envelopment analysis (DEA) was proposed by Charnes and Cooper (1978) on the basis of the concept of “relative efficiency evaluation” and is applicable to the evaluation of the efficiency of decision units with multiple inputs and outputs [37], but it ignores the measurement error caused by slack variables. To overcome this problem, Tone (2001) introduced a non-radial and non-angle SBM model based on the slack measure, which solved the problems of weak accuracy and slackness of the traditional DEA model; however, the model could not be used for comparative analysis of multiple decision units with an efficiency value of 1 [38]. Based on this, Tone (2002) proposed a super-efficiency SBM model combining super-efficiency and SBM model [39]. The SBM model adds non-desired output variables and corrects the slack variables and, at the same time, is able to decompose multiple decision units with an efficiency value of 1. Therefore, the SBM model can compare and rank effective decision units, improving its practical applicability. For this reason, this study used the super-efficiency SBM model to measure the CEE of cities in the YRB, and the mathematical expression is:

(1) $$\min\rho =\frac{\frac{1}{m}\sum_{p=1}^{m}\frac{{\bar{x}}_{i}}{x_{i0}}}{\frac{1}{S_{1}+S_{2}}(\sum_{q=1}^{S_{1}}\frac{{\bar{y}}_{q}^{w}}{y_{q0}^{w}}+\sum_{q=1}^{S_{2}}\frac{{\bar{y}}_{q}^{b}}{y_{q0}^{b}}}\;s.t.\left\{\begin{array}{c} \begin{array}{c} \bar{x}\geq \sum_{j=1,\neq k}^{n}\theta_{j}x_{j}\\ \bar{y^{w}}\leq \sum_{j=1,\neq k}^{n}\theta_{j}y_{j}^{w}\\ \bar{y^{b}}\geq \sum_{j=1,\neq k}^{n}\theta_{j}y_{j}^{b}\\ \bar{x}\geq x_{0},0\leq \bar{y^{w}}\leq y_{0}^{w},\bar{y^{b}}\geq y_{0}^{b},\theta \geq 0 \end{array} \end{array}\right.\;$$

where *ρ* is the measured efficiency value, and a higher value indicates higher relative efficiency of the decision unit; *m, S*_1_, *S*_2_ denote the number of input indicators, desirable and undesirable output; $x_{i0},\;y_{q0}^{w},\;y_{q0}^{b}\;$ denote input indicators, desirable and undesirable output indicators, respectively, and $x_{i},y_{q}^{w},y_{q}^{b}$ denote the slack of input, desired and non-desired output, respectively.

The study establishes a CEE indicator system from three dimensions: economic development level, labor force, and environmental status (Table 1). Among them, the input indicators specifically include the capital factor, labor factor, and energy [31]. For the capital factor, the fixed capital stock was measured by referring to the perpetual inventory method, and the corresponding fixed asset investment price index was selected and deflated using 2003 as the base period; The labor and energy factors were characterized by the number of employees and total annual electricity consumption respectively, where the number of employees was obtained by summing up the number of urban unit employees and the number of urban private and self-employed employees at the end of the period. The desirable and undesirable output indicators were the gross regional product and carbon dioxide emissions, respectively, where the gross regional product was deflated according to the GDP deflator and expressed as the real value of the gross regional product.

### 2.3. Theil Index Decomposition

The Theil index is an important indicator of regional socio-economic differences and was first proposed by Theil [40]. The Theil index has good decomposability. When the sample is divided into multiple clusters, the Theil index can be decomposed into between-group disparities and within-group disparities. In this paper, based on the basic idea of the Theil index decomposition, the YRB was divided into two categories of resource and non-resource-based cities, and the Theil index of regional differences in CEE in the YRB was constructed. The relevant measures and decomposition formulas are shown in Equations (2)–(4).

(2) $$T=T_{b}+T_{w}$$

(3) $$T_{b}=\overset{K}{\underset{k=1}{\sum }}y_{k}\ln\left(\frac{y_{k}}{n_{k}/n}\right)$$

(4) $$T_{w}=\overset{K}{\underset{k=1}{\sum }}y_{k}(\underset{i\in g_{k}}{\sum }\frac{y_{i}}{y_{k}}\ln\left(\frac{y_{i}/y_{k}}{1/n_{k}}\right)\;$$

where *T* represents the Theil index of the overall gap of CEE in the YRB; $T_{w}$ and $T_{b}\;$ represent the intra-regional gap and inter-regional gap of CEE in the YRB, respectively; *n* represents the number of regions; $y_{i}$ represents the share of CEE of individual *i*, and $y_{k}$ represents the share of CEE of group *k*. The larger the value of the Theil index, the greater the degree of difference in CEE among regions, and vice versa.

### 2.4. Panel Data Regression Model

IPAT, firstly proposed by Ehrlich and Holdren, is a quantitative relational model for studying the impact of human activities on the environment, arguing that environmental change is the result of a combination of population increase, economic growth, and technological progress [41]. However, the IPAT model simplifies the relationship between each influencing factor and the resource-environment; in fact, the relationship between each influencing factor and the resource-environment is often not a simple linear relationship. To overcome the limitations such as the homogeneous linear relationship between environmental impacts and each driver, Dietz et al. (1994) proposed the nonlinear stochastic regression impact model [42]. The Stochastic Impacts by Regression on Population, Affluence, and Technology (STIRPAT) model is an extensible stochastic environmental impact assessment model, which, by assessing the relationship between the three independent and dependent variables of population, property, and technology, can be used to analyze the non-proportional impact of economic and social factors on the environment.

Based on the influence of technology, economy, and population on CEE, the basic expression of the STIRPAT model is derived as:

(5) $$I=\mu T^{a}A^{b}P^{c}\varepsilon$$

where *T*, *A*, and *P* denote technological innovation, affluence, and population size, respectively; *μ* and *ε* are the constant term and random error term, respectively. To reduce the effect of heteroskedasticity, Equation (5) is transformed into a logarithmic form as follows:

(6) lnI=μ+alnT+blnA+clnP+ε

Factors such as the level of economic development, industrial structure, green technological innovation, and population density are incorporated into the STIRPAT model as follows:

(7) $$\ln CEE_{mn}=\mu_{0}+\mu_{1}\ln ED_{mn}+\mu_{2}\ln IS_{mn}+\mu_{3}\ln GTI_{mn}+\mu_{4}\ln PD_{mn}+\mu_{5}\ln FDI_{mn}+\mu_{6}\ln RE_{mn}+\ln\varepsilon$$

where *CEE*, *ED*, *IS*, *GTI*, *PD*, *FDI*, and *RE* denote carbon emission efficiency, economic development level, industrial structure, green technological innovation, population density, foreign investment intensity, and resource endowment, respectively; *μ*_1_, *μ*_2_, *μ*_3_, *μ*_4_, *μ*_5,_ and *μ*_6_ are their corresponding elasticity coefficients, which denote the change in *CEE* when *ED*, *IS*, *GTI*, *PD*, *FDI*, and *RE* change by 1% respectively; *m* is the city, *n* is the year and *μ*_0_ is a constant.

## 3. Results

### 3.1. Temporal Evolution Characteristics of CEE

**Figure 2:**
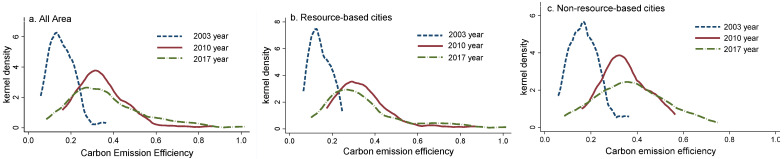
Kernel density distribution of CEE.

Considering that 2010 was a turning point between the Eleventh and the Twelfth Five-Year Plan period, this study selected cross-sectional data for the time periods of 2003, 2010, and 2017. The kernel density curves of the YRB and its resource and non-resource-based cities are shown in Figure 2.

(1) Time evolution of CEE of cities in the whole region. From the position of the curve, the kernel density estimation curve shows a rightward shift, indicating that the CEE of cities in the YRB has maintained an overall upward trend during the study period; the kernel density estimation curve widens over time and the peak gradually decreases, as the width gradually becomes wider, the range of interval variation increases, indicating an increase in the regional differences in urban CEE; all three curves show a single peak, without bipolar or multi-polar differentiation; the right trailing edge of the curve is longer than the left trailing edge every year and shows a lengthening trend, which indicates that some cities have lower efficiency. The cities in the high-value area account for a lower proportion than those in the low-value area, and the higher the efficiency value, the lower the proportion of cities.

(2) Time evolution of CEE in resource and non-resource-based cities. The kernel density estimation curves of both types of cities show a trend of rightward shift, but resource-based cities shift slightly to the left after the rightward shift, while the rightward shift of non-resource-based cities continues to increase; in terms of distribution pattern, the curves of both types of cities show a gradual decrease in peak value and widening, while the change interval of resource-based cities is larger and the change interval of non-resource-based cities is relatively smaller, which indicates that the absolute difference tends to expand in both types of cities, and the absolute difference is greater in resource-based cities; the trailing on the right side of the curve is longer than the trailing on the left side for both types of cities each year, and the trailing on the right side shows a lengthening and decreasing trend; the trailing on the right side is longer for resource-based cities. Overall, resource-based cities mainly show the characteristics of decentralized regional agglomeration, while non-resource-based cities mainly show the characteristics of convergent regional agglomeration.

### 3.2. Spatial Evolution Characteristics of CEE

From an overall perspective, the Thiel index of CEE in the YRB shows a “U” pattern, and the regional differences show a trend of convergence and then expansion (Table 2). From the decomposition results of the Theil index, the regional differences of CEE in the YRB mainly come from the differences between resource cities and non-resource-based cities. In contrast, the variability between the two types of cities is relatively small. From the intra-group differences of different regions, the intra-group differences of CEE of resource and non-resource-based cities show a widening trend. The internal imbalance of resource-based cities is more severe. Resource-based cities should formulate corresponding development strategies, such as increasing investments in fixed assets and innovation factors, optimizing the industrial structure and employment structure to reduce resource dependence. At the same time, due attention should be paid to the balanced development of the YRB as a whole, encouraging the implementation of innovation-driven development strategies in the YRB, and optimizing industrial and energy structures, promoting the incubation and transformation of science and innovation achievements in resource-based cities, strengthening inter-regional cooperation and exchange, and forming a regional economic layout with complementary advantages and high-quality development.

**Figure 3:**
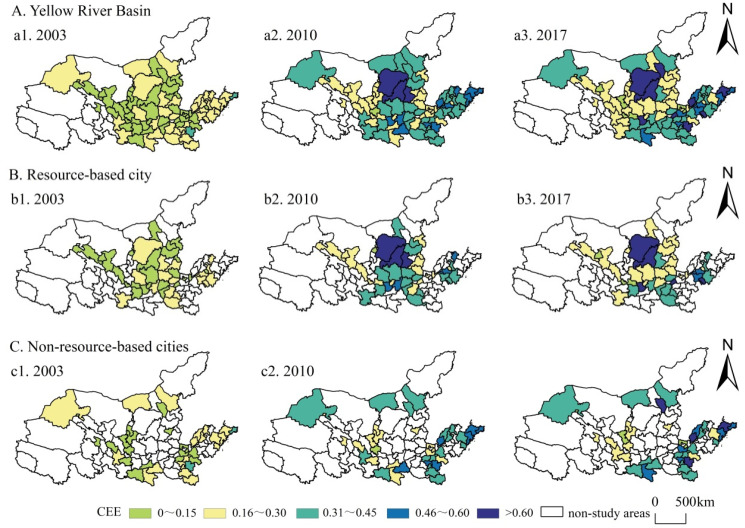
Spatial distribution of CEE in the YRB.

The study selected cross-sectional data for three time periods 2003, 2010, and 2017, and divided CEE into five classes. Areas with efficiency values ≤0.15 were considered low-value areas for CEE, 0.16~0.30 lower-value areas, 0.31~0.45 medium-value areas, 0.46~0.60 higher-value areas, and ≥0.6 high-value areas. The spatial pattern of CEE in the YRB and its resource and non-resource-based cities is shown in Figure 3.

(1) Spatial evolution of cities in the whole region. The cities in the YRB present a spatial differentiation in CEE, with an overall distribution pattern high in the east and south and low in the west and north. There is a spatial clustering among cities with higher and lower CEE. Specifically, Qingdao, Yantai, and Weihai in eastern Shandong Province and Erdos in Inner Mongolia have relatively high CEE. These regions have a better foundation of economic development and have high output efficiency but also have a more sustainable industrial structure, upholding the concept of ecological civilization and sustainable development and paying attention to the high-quality development of the economy. In contrast, Jinchang City in Gansu Province and Datong and Yangquan in Shanxi Province have relatively low CEE. Most of these cities are resource-based. Their industrial production emits a large number of pollutants, coupled with a low level of technological innovation. Environmental supervision is not in place, the ecological civilization system has not been established, and there are difficulties in their low-carbon transition development.

(2) Comparison of spatial evolution between resource and non-resource-based cities. The number of resource-based cities in the high-value CEE zone increased while the number of those in the low-value zones decreased from 2003 to 2017. Most cities were at the lower and middle-level stages of CEE in 2017, and the cities in the high-value CEE zone included Erdos, Yulin, Zaozhuang, Xianyang, and Jiaozuo City. Most non-resource-based cities have improved their CEE levels, and most of them are in the middle and higher-level states of CEE. Non-resource-based cities in the eastern coastal region have higher CEE, and the high-value areas in 2017 are distributed in Yantai and Liaocheng in Shandong Province, Hohhot in Inner Mongolia, and Zhoukou in Henan Province. Taken together, the overall CEE level of non-resource-based cities was higher than that of resource-based cities in 2017, and the divergence between resource-based cities was obvious.

### 3.3. Analysis of Factors Influencing CEE

#### 3.3.1. Variable Selection

The study selected CEE as the response variable and used economic development level, industrial structure, green technological innovation, population density, foreign investment intensity, and resource endowment as explanatory variables to explain CEE in the YRB in a comprehensive manner (Table 3).

Response variable: carbon emission efficiency (*CEE*). *CEE* has the characteristics of an “all-factor”, which is the result of the combined effect of material input, labor consumption, and economic development.

Explanatory variables: economic development level (*ED*), measured as GDP per capita, was used to indicate the influence of urban affluence on CEE [27]; industrial structure (*IS*) is an essential influencing component of CEE [43], the dominant industry in some cities of the YRB is single, and the excessive share of secondary industry, mainly heavy industry, is one of the important causes of carbon emission; green technological innovation (*GTI*), the economic strength of enterprises and research institutions and the knowledge level of researchers are used to develop new technologies and apply the existing ones [26]. This study used the sum of green patents and green utility model patents reported in the “International Patent Classification Green List” by the World Intellectual Property Organization (WIPO) to represent *GTI*; population density (*PD*), the agglomeration and scale effect brought by the increase of population density in the urbanization process may affect carbon emissions by improving the efficiency of resource use [44]; foreign direct investment (*FDI*), the ratio of actual foreign capital use to GDP, was used to measure the impact of the degree of openness on CEE [45]. This study used the proportion of extractive industry employees to the total population at the end of the year to measure resource endowment (*RE*) [46].

#### 3.3.2. Fitting Analysis of CEE and Related Variables

**Figure 4:**
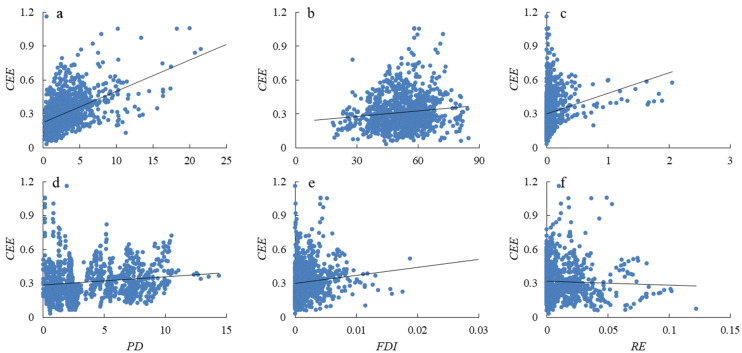
Correlation analysis between explanatory variables and CEE. (**a**) Scatter plot fitting of economic development (*ED*) and CEE; (**b**) Scatter plot fitting of industry structure (*IS*) and CEE; (**c**) Scatter plot fitting of green technology innovation (*GTI*) and CEE; (**d**) Scatter plot fitting of population density (*PD*) and CEE; (**e**) Scatter plot fitting of foreign investment intensity (*FDI*) and CEE; (**f**) Scatter plot fitting of resource endowment (*RE*) and CEE.

The study fitted scatter plots to each explanatory variable selected by the model separately with CEE (Figure 4). Initially, it is determined that there is a certain correlation between the level of economic development (*ED*), industrial structure (*IS*), green technology innovation (*GTI*), population density (*PD*), foreign investment intensity (*FDI*), resource endowment (*RE*), and *CEE*. To investigate the influence mechanism of each influencing factor on CEE, we need to further develop a model to clarify the direction and coefficient of each variable.

#### 3.3.3. Descriptive Statistics and Stability Test

The descriptive statistical results of the indicator data are shown in Table 4. This study used the ADF and LLC methods to conduct unit root tests on the variable data and ensure the validity of regression results. Table 5 shows that the test data are all stable and there is no unit root.

#### 3.3.4. Analysis of Full-Sample Regression Results

A panel quantile regression model was established based on the regional gradient of CEE, and five representative quantile points of 10%, 25%, 50%, 75%, and 90% were selected to explore the non-linear effects of different influencing factors on CEE. The regression results are presented in Table 6.

The economic development level is significantly and positively correlated with the CEE in the YRB, passing the significance test at a 1% confidence level at all quantiles. The estimated coefficients increase with the increase of quantiles and reach the maximum value at 90% quantile, indicating that the enhancement of the economic strength of cities can drive the improvement of CEE in the YRB. The influence is stronger in areas with higher CEE. Regions with higher levels of economic development are better able to increase capital investment related to carbon emission reduction. They promote CEE by setting up special funds for energy conservation, emission reduction, and carbon reduction, supporting technological research, and development in emerging fields such as advanced energy storage, clean production, and renewable energy and promoting the development of an advanced, and environmentally-friendly industrial structure.

At present, the main characteristics of the industrial structure of some cities in the YRB are the unevenness of development, the low proportion of tertiary industries, and the high proportion of traditional resource-intensive industries, and the prominent problems of high energy consumption, high water consumption, and high emissions. For different quantiles of CEE conditions, the influence coefficients of industrial structure and CEE are negative in the lower quantiles (10% and 25%) and positive in the other quantile. Their absolute values increase gradually with the increase of quantiles.

Some scholars believe that technological innovation can improve the level of low-carbon technology and energy resource utilization efficiency, which in turn improves CEE [47]. But at the same time, technological innovation stimulates economic activities and brings more energy consumption and carbon emissions, which may offset the energy saved due to efficiency improvement. The empirical results of this paper show the complexity of the effect of technological innovation on CEE, with technological innovation showing a significant negative effect in the high quantiles (75% and 90%) and a significant positive effect in the low quantile (10%), which indicates that technological innovation is the main emission reduction factor in regions with low CEE, and that reducing carbon through alternative clean energy, carbon capture, and storage technologies, and energy efficiency improvements can effectively improve CEE; in contrast, in regions with higher CEE, there is a negative correlation, probably because the emission reduction effect brought by technological efficiency improvements cannot offset the carbon emission growth effect brought by its promotion of economic growth.

Population density, an important population factor, affects CEE mainly by influencing lifestyle behaviors such as public transportation, resource use, and sharing of pollution control and emission reduction facilities with a two-sided effect [24]. On the one hand, the scale and agglomeration effects of increasing population density are conducive to the concentration of highly qualified talents and high-end production factors, thus promoting low-carbon transformation and high-quality development of cities and improving CEE. On the other hand, the rapid concentration of population in cities and high population density can lead to environmental degradation, traffic congestion, and insufficient supply of public services. The empirical results show that population density can significantly improve the efficiency of urban carbon emissions, and no significant diseconomies of agglomeration emerge.

The relationship between foreign direct investment (FDI) and carbon emissions is generally studied in the context of the “Pollution Heaven Hypothesis” [48] and “Pollution Halo Hypothesis” [49]. The empirical results of this paper show that FDI is negatively correlated with CEE, which further supports the possibility of the “Pollution Heaven Hypothesis”, while the “Pollution Halo Hypothesis” suggests that the technology spillover effect of FDI on the host country is relatively limited, indicating that there is still some room for improvement in the structure and efficiency of foreign investment.

Resource endowment is significantly negatively correlated with CEE and shows a stronger inhibitory effect on areas with higher CEE. The possible explanations for this are: the greater intensity of resource development in some areas of the YRB, the scale effect and agglomeration effect brought by it attracts more enterprises to join the resource development in the region, which easily leads to resource path dependence, resulting in the repeated construction of high energy-consuming, high-polluting, and high-emission projects, the lagging development of successive alternative industries, the high proportion of heavy industry and the single industrial structure, which are not conducive to the improvement of urban CEE.

#### 3.3.5. Comparison of Factors Influencing CEE between Resource and Non-Resource-Based Cities

The study dissected the influencing factors of CEE in 41 resource-based cities in the YRB and introduced 38 non-resource-based cities to compare and analyze the differences between the two types of cities. Regressions were conducted using the random-effects model (REM) and fixed-effects model (FEM), respectively. The regression results of the fixed-effects model were selected in both types of cities in combination with the Hausman test results (Table 7).

(1) Resource-based cities. Except for population density, which does not pass the significance test, all variables are significantly and positively correlated with CEE. This indicates that multiple factors need to work together in the low-carbon transformation of resource-based cities, improving economic development and green technological innovation, optimizing industrial structure, promoting foreign direct investment, and highlighting the advantages of resource endowment all have a significant contribution to the CEE of resource-based cities. Due to objective factors such as the improvement of environmental protection requirements in the YRB in recent years, the proportion of industry in resource-based cities in the basin has decreased. However, there is still a high proportion of secondary industry. At the same time, the leading industries in resource-based cities are mainly equipment manufacturing and traditional energy chemical factories. The resource-based cities should adjust their industrial structure according to their location advantages, resource endowment, and industrial characteristics, strengthen technological innovation, accelerate the deep integration of new-generation information technology and industry, enhance the independent innovation capability of industrial enterprises, and gradually improve their opening-up capability.

(2) Non-resource-based cities. The regression coefficients of GDP per capita and industrial structure are positive and pass the 1% confidence level test, indicating that the improvement of economic development level and the upgrading of industrial structure promote the CEE of non-resource cities; the regression coefficient of FDI is negative and passes the 5% confidence level test, indicating that foreign direct investments have a suppressive effect on the improvement of CEE in non-resource-based cities, which supports the possibility of the “Pollution Heaven Hypothesis”. Green technological innovation, population density, and resource endowment did not pass the significance test. The results indicate that the level of economic development and industrial structure optimization are the main driving forces in the process of low-carbon transformation of non-resource-based cities. The future needs to accelerate the transformation of foreign investment structure, adjust the balanced relationship between foreign investment and foreign opening, give full play to the important role of foreign investment in technological innovation and industrial transformation and upgrading, and gradually improve the efficiency of carbon emissions.

(3) Comparison results. Commonality: the level of economic development and industrial structure significantly promote the CEE of both resource and non-resource-based cities in the YRB. Difference: green technological innovation has a significant positive effect on CEE in resource-based cities, and the effect on non-resource-based cities is not significant. This indicates that within resource-based cities, the emission reduction effect brought by technological innovation through new technologies, equipment, and processes to improve the efficiency of production factors and energy use promotes CEE, while for non-resource-based cities, the final impact effect is insignificant and may be due to the fact that the emission reduction effect of technological innovation is offset by the carbon emission growth effect of its driving economic development. The significant promoting effect of FDI on CEE in resource-based cities and the significant inhibiting effect on CEE in non-resource-based cities suggest that FDI improves energy use efficiency and CEE in resource-based cities through knowledge and technology spillover, while for non-resource-based cities, it may be due to the relatively limited technology spillover effect of FDI on non-resource-based cities. Resource endowment promotes carbon efficiency in resource-based cities, not significant for non-resource-based cities.

## 4. Conclusions and Recommendations

### 4.1. Conclusions

The study used the Super-SBM model to measure the CEE of cities in the YRB, and compared the spatial and temporal evolution characteristics and influencing factors of the YRB and its resource and non-resource-based cities, drawing the following conclusions.

Firstly, in terms of temporal evolution, the CEE of the YRB as a whole and its resource and non-resource-based cities show an increasing trend, and the nuclear density curve flattens and widens. The regional disparity of urban CEE shows a widening trend. Resource-based cities are mainly characterized by decentralized regional agglomeration, while non-resource-based cities are mainly characterized by convergent regional agglomeration.

Secondly, in terms of spatial evolution characteristics, the Theil index of CEE in the YRB shows a “U” pattern, first decreasing and then increasing. The decomposition of the Theil index shows that the regional differences in CEE mainly come from the differences within resource and non-resource-based cities; the CEE of cities in the YRB shows obvious spatial differences, with a distribution pattern of high in the east and south and low in the west and north. There is an increase in the number of high-value areas and a decrease in the number of low-value areas, and obvious spatial clustering characteristics of cities with higher and lower CEE.

Thirdly, from the model estimation results of the YRB as a whole, the level of economic development, industrial structure, and population density are significantly positively correlated with CEE in the YRB, and foreign direct investment and resource endowment are significantly negatively correlated with CEE. Green technological innovation has a significant negative effect on CEE in the high quantiles (75% and 90%) and shows a significant positive effect in the lower quantile (10%).

Fourthly, comparison of the factors influencing CEE in resource and non-resource-based cities in the YRB. Commonality: economic development level and industrial structure have significant promoting effects on the CEE of both types of cities. Difference: technological innovation has a significant positive effect on the CEE of resource-based cities, while the correlation with non-resource-based cities does not pass the test; foreign direct investment has a significant promoting effect on the CEE of resource-based cities and a significant inhibiting effect on the CEE of non-resource-based cities. Resource endowment has a significant positive correlation with the CEE in resource-based cities and insignificant for non-resource-based cities.

Based on the analysis of the spatiotemporal evolution characteristics and influencing factors of CEE in the YRB, this study combines differences in urban resource dependency to conduct a typological partitioning of carbon emission reduction mechanism research in the YRB, strengthening the comparative study of similarities and differences in CEE between resource-based and non-resource-based cities, and systematically explore carbon emission and carbon reduction paths in different types of cities in the process of low-carbon transition. The research framework of this study can be applied to other regions, and it provided a reference for predicting and modeling the effects of urban carbon reduction and developing government policies.

### 4.2. Recommendations

The low-carbon transition in the YRB is of great significance for China to achieve the “double carbon” target. Based on the above findings, this study proposes the following countermeasures. The relevant departments of the State Council indicated that the YRB should take ecological protection as the goal, science, and technology as the support, and information technology as the means, correctly handle the relationship between the development of strategic new industries and the transformation and upgrading of traditional industries, use the Internet, artificial intelligence, big data and other advanced technologies to upgrade the traditional industrial sectors, further promote the YRB advantageous manufacturing industry green, intelligent, digital. In addition, local people’s governments at all levels implement zoning and classification policies to promote coordinated regional development. Due to the large differences in economic development level and resource endowment in different regions, each city in the YRB should take into account its own situation and develop a low-carbon development model suitable for the region. For example, some highly industrialized cities can vigorously introduce or cultivate leading enterprises to create competitive new industrial clusters. Enterprises should prioritize the use of clean energy and adopt processes and tools with high resource utilization and low carbon emissions as well as comprehensive waste utilization technologies to reduce the generation of carbon emissions. Resource-based cities should reduce the dependence of economic growth on resources while developing mineral resources rationally, improve the efficiency of resource utilization, gradually form a regional layout of high-quality development in the YRB with integrated development and complementary advantages, and promote coordinated regional development.

To implement the national major strategy of environmental protection and high-quality development in the YRB, the Ministry of Ecology and Environment and other 12 departments have jointly issued the Action Plan for *the Battle of Yellow River Ecological Protection and Treatment* in 2022. The action plan highlights the importance of environmental protection and low-carbon development in the YRB. We will further strengthen the research related to CEE in the YRB. On the basis of adopting total indicators, it will continue to optimize the carbon emission efficiency input-output indicator system by adopting relative indicators (e.g., per capita indicators, land-average indicators, or intensity indicators) to measure CEE. Due to data availability, this study chose the total annual electricity consumption to measure urban energy consumption. However, in the future, we will use big data to reflect the energy consumption of each city to improve the accuracy of CEE. Additionally, there may be spatial spillover effects between regions for the influencing factor variables. Therefore, we will use a spatial econometric model to study the spatial spillover effect of each influencing factor on CEE in subsequent studies.

## Figures and Tables

**Table 1:** System of CEE input-output index.

Indicator Type	Primary Indicators	Secondary Indicators	Unit
Input Indicators	Capital factor	Fixed capital stock	10^8^ yuan
	Labor factor	Number of employees	10^4^ people
	Energy factor	Total annual electricity consumption	10^4^ kW·h
Output Indicators	Desirable output	GDP	10^8^ yuan
	Undesirable output	Total Carbon Emissions	10^4^ t

**Table 2:** Decomposition of the Theil index of CEE.

Year	Resource-Based Cities	Non-Resource-Based Cities	Intra-Group Variation	Inter-Group Variation	Total
2003	0.0266	0.0470	0.0736	0.0034	0.0770
2008	0.0435	0.0279	0.0710	0.0009	0.0719
2010	0.0379	0.0213	0.0592	0.0001	0.0594
2013	0.0442	0.0313	0.0756	8.5 × 10^−7^	0.0756
2017	0.0660	0.0477	0.1137	0.0002	0.1138

**Table 3:** Description of the variables used in the model.

Variables	Indicators	Definition
Response variable	Carbon Emission Efficiency	measured by super-efficiency SBM model
Explanatory variables	Economic Development	Real GDP per capita
	Industry Structure	Value added of secondary industry/GDP
	Green Technology Innovation	Number of green invention patents + utility model patents granted
	Population density	Total population/municipality area
	Foreign Direct Investment	Actual utilization of foreign capital/GDP
	Resource Endowment	The proportion of employees in the extractive industry to the total population at the end of the year

**Table 4:** Descriptive statistics of variables.

Variables	Unit	Mean	Std. Dev.	Min	Max
*CEE*	—	0.3159	0.1557	0.0306	1.1606
*ED*	10^4^ yuan	3.4482	3.0456	0.1892	25.6877
*IS*	%	51.5921	11.9185	9	84.88
*GTI*	10^4^ pieces	0.0086	0.0224	0	0.235
*PD*	10^4^ persons/sq.km	0.3934	0.0305	0.0005	0.1440
*FDI*	dollars/yuan	0.0021	0.0030	1.05 × 10^−11^	0.0341
*RE*	%	1.12	1.69	8.04 × 10^−6^	12.21

**Table 5:** Stability test of panel data.

Variables	LLC		ADF		Conclusion
	Statistics	*p*-Value	Statistics	*p*-Value	
*CEE*	−7.6187	0.0000	3.1608	0.0008	Stable
*ED*	−1.7855	0.0371	3.9194	0.0000	Stable
*IS*	−5.5630	0.0000	9.4323	0.0000	Stable
*GTI*	−2.4582	0.0070	1.6933	0.0452	Stable
*PD*	−4.0735	0.0000	11.3532	0.0000	Stable
*FDI*	−1.5604	0.0593	2.0803	0.0187	Stable
*RE*	−2.8669	0.0021	4.9822	0.0000	Stable

**Table 6:** Quantile regression estimation results.

Variables	q10	q25	q50	q75	q90
*ED*	0.0167 ***	0.0177 ***	0.0309 ***	0.0383 ***	0.0473 ***
	(8.74)	(8.43)	(13.44)	(10.12)	(12.69)
*IS*	−0.0003	−0.0004	0.0004	0.0010	0.0012 *
	(−0.79)	(−1.50)	(1.18)	(1.56)	(1.92)
*GTI*	0.4452 ***	0.1881	−0.1938	−0.5841 ***	−1.5998 ***
	(2.70)	(1.13)	(−1.24)	(−2.74)	(−6.88)
*PD*	1.1399 ***	0.9636 ***	0.6364 ***	0.5563 ***	1.0000 ***
	(8.67)	(9.43)	(7.15)	(2.69)	(3.04)
*FDI*	−1.0341	1.5592	−1.1146	−4.8099 **	−1.7909
	(−0.75)	(0.94)	(−0.76)	(−2.30)	(−0.34)
*RE*	−0.5013 ***	−0.6929 ***	−1.3663 ***	−2.2521 ***	−2.5186 ***
	(−3.10)	(−4.56)	(−9.01)	(−8.71)	(−5.61)
*Cons*	0.0963 ***	0.1512 ***	0.1633 ***	0.2081 ***	0.2675 ***
	(7.91)	(15.45)	(10.09)	(7.54)	(8.16)

Note: ***, ** and * represent 1%, 5% and 10% significance levels, respectively.

**Table 7:** Results of regression estimation of variables.

Variables	Resource-Based Cities		Non-Resource-Based Cities	
	REM	FEM	REM	FEM
*ED*	0.0278 ***	0.0283 ***	0.0402 ***	0.0418 ***
	(14.08)	(14.05)	(20.83)	(21.63)
*IS*	0.0036 ***	0.0043 ***	0.0048 ***	0.0056 ***
	(6.02)	(6.91)	(8.89)	(10.22)
*GTI*	3.5329 ***	3.5093 ***	0.1094	0.1320
	(4.31)	(4.26)	(0.67)	(0.83)
*PD*	0.2838	1.4387	0.8421 **	0.8877
	(0.50)	(1.44)	(2.22)	(1.61)
*FDI*	2.7303	3.0568 *	−2.4949 **	−2.2916 **
	(1.49)	(1.66)	(−2.33)	(−2.18)
*RE*	0.5488	1.7877 ***	−0.1692	0.0259
	(1.02)	(2.80)	(−0.32)	(0.05)
*Cons*	−0.0244	−0.1275 ***	−0.0753 **	−0.1233 ***
	(−0.59)	(−2.62)	(−2.29)	(−3.64)
*R^2^*	0.4360	0.4419	0.6214	0.6226
*F*-statistic	—	20.27	—	40.00

Note: ***, ** and * represent 1%, 5% and 10% significance levels, respectively.

## Data Availability

The data that support the findings of this study are available upon request from the corresponding author.
